# Roles of PI3K and JAK pathways in viability of retinal ganglion cells after acute elevation of intraocular pressure in rats with different autoimmune backgrounds

**DOI:** 10.1186/1471-2202-9-78

**Published:** 2008-08-11

**Authors:** Yao Huang, Zhiwei Li, Ningli Wang, Nico van Rooijen, Qi Cui

**Affiliations:** 1 Department of Ophthalmology and Visual Sciences, The Chinese University of Hong Kong, Hong Kong, P.R. China; 2 Department of Ophthalmology, Beijing Tongren Hospital, Capital University of Medical Sciences, Beijing, P.R. China; 3 Department of Cell Biology and Immunology, Faculty of Medicine, Vrije Universiteit, 1081 BT Amsterdam, The Netherlands

## Abstract

**Background:**

We recently showed that whereas inhibition of PI3K/akt or JAK/STAT pathway promoted retinal ganglion cell (RGC) survival after optic nerve (ON) injury in Fischer 344 (F344) rats, the same inhibition resulted in aggravated RGC loss after acute intraocular pressure (IOP) elevation in Sprague Dawley (SPD) rats. In addition, the responses of macrophages to ON injury and acute IOP elevation were different between F344 and Lewis rats, i.e., different autoimmune profiles. Using an acute IOP elevation paradigm in this study, we investigated 1) whether autoimmune background influences PI3K/akt and JAK/STAT functions by examining the effect of PI3K/akt and JAK/STAT pathway inhibition on RGC survival in F344 and Lewis rats, and 2) whether differential actions of macrophages occur in PI3K/akt and JAK/STAT pathways-dependent modulation of RGC survival. IOP elevation was performed at 110 mmHg for 2 hours. PI3K/akt and JAK/STAT pathway inhibitors were applied intravitreally to block their respective pathway signaling transduction. Because macrophage invasion was seen in the eye after the pathway inhibition, to examine the role of these pathways independent of macrophages, macrophages in the retina were removed by intravitreal application of clodronate liposomes. Viable RGCs were retrogradely labelled by FluoroGold 40 hours before animal sacrifice.

**Results:**

Similar to what was previously observed, significantly more RGCs were lost in Lewis than F344 rats 3 weeks after acute IOP elevation. As in SPD rats, inhibition of the PI3K/akt or JAK/STAT pathway increased the loss of RGCs in both F344 and Lewis rats. Removal of macrophages in the eye by clodronate liposomes reduced RGC loss due to pathway inhibition in both strains.

**Conclusion:**

This study demonstrates that following acute IOP elevation 1) PI3K/akt and JAK/STAT pathways mediate RGC survival in both F344 and Lewis rats, 2) autoimmune responses do not influence the functions of these two pathways, and 3) PI3K/akt and JAK/STAT pathway inhibition-dependent activation of macrophages is detrimental to RGCs.

## Background

Loss of retinal ganglion cells (RGCs) occurs in many pathological situations, and glaucoma is one of the common diseases that lead to RGC loss. A common feature of glaucoma is elevation of intraocular pressure (IOP) that causes progressive axonal degeneration and loss of RGCs. As acute glaucoma is characterised by rapid increase in IOP, acute IOP elevation paradigm in rodents has often been used to study acute IOP elevation-induced retinal ischemic/reperfusion injury and the possible mechanisms underlying acute glaucoma-associated RGC injuries [1-5].

It is known that immune responses can influence neuronal survival after injury. Whereas ample evidence pointed to a damaging effect of inflammatory responses of macrophages and T-cells after CNS injury and in autoimmune diseases such as multiple sclerosis and experimental autoimmune encephalomyelitis (EAE) [6-13], T-cell dependent RGC protection in response to IOP elevation has also been reported in EAE-resistant Fischer 344 (F344) but not EAE-vulnerable Lewis rats [14]. In our earlier study, we found that macrophages, which are another major component of the autoimmune system, responded differently to IOP elevation or optic nerve (ON) injury between F344 and Lewis rats, and such differences led to different extents of RGC survival after IOP elevation or ON injury [15,16]. Macrophage activation in the eye by intravitreal injection of zymosan, a yeast wall preparation, protected RGCs after ON injury whereas the same activation aggravated RGC loss following acute IOP elevation [15-17]. In addition, under the same condition of either ON injury or acute IOP elevation the same macrophage activation resulted in different extents of RGC survival or loss between F344 and Lewis rats [16,17].

Phosphatidylinositol 3-kinase (PI3K)/akt and janus kinase (JAK/STAT3) signal pathways are well known to mediate neuronal survival [18-25]. Recently we showed that ON injury or acute IOP elevation activates both pathways in the ganglion cell layer (GCL) [26-28]. However, our recent work also points to paradoxic actions of these two pathways in RGC survival under different pathological conditions. Though both ON injury and acute IOP elevation activate PI3K/akt and JAK/STAT pathways in the GCL, including RGCs, inhibition of these signalling pathways activates macrophages in the eye and contributes to RGC survival after ON injury in F344 rats [28]. However, the same pathway inhibition leads to RGC loss following acute IOP elevation in Sprague Dawley (SPD) rats [26,27]. In addition, as mentioned above, we also showed that macrophage activation in the eye by intravitreal injection of zymosan played a protective role in RGCs after ON injury [15,28]. But the same macrophage activation resulted in aggravated RGC loss in an IOP elevation model of the same F344 rat strain [17]. Macrophages thus appear to play an important role in the differences in RGC viability under the two pathological conditions.

F344 and Lewis rats are inbred strains. They differ in susceptibility to EAE. It is known that the hypothalamic-pituitary-adrenal (HPA) axis modulates autoimmune response and vulnerability to EAE. In contrast to F344 rats, Lewis rats have abnormalities in HPA function. Variation in genotypes that are associated with different disease phenotypes between F344 and Lewis rats have been reported [29]. Furthermore, the greater frequency of CD8^+ ^regulatory T cells, which functionally inhibit myelin basic protein-reactive T-cells, in F344 than in Lewis rats might contribute to the differing susceptibility to EAE between them [30]. It is currently unknown whether, after acute IOP elevation, 1) inhibition of the PI3K/akt or JAK/STAT pathway aggravates RGC loss in F344 and Lewis rats, 2) autoimmune background influences PI3K/akt and JAK/STAT signaling pathways in RGC survival, and 3) differential actions of macrophages occur in PI3K/akt and JAK/STAT pathway-dependent modulation of RGC survival. PI3K/akt and JAK/STAT pathways play an important role in mediating RGC survival following acute IOP elevation in SPD rats [26,27]. Autoimmune background is also known to modulate neuronal viability [16,17]. It is therefore important to clarify the issues as mentioned above. This study, using F344 and Lewis rats, was designed to accomplish these tasks.

## Results

### Effect of inhibition of PI3/akt and JAK/STAT pathways on RGC viability in F344 rats

**Figure 1:**
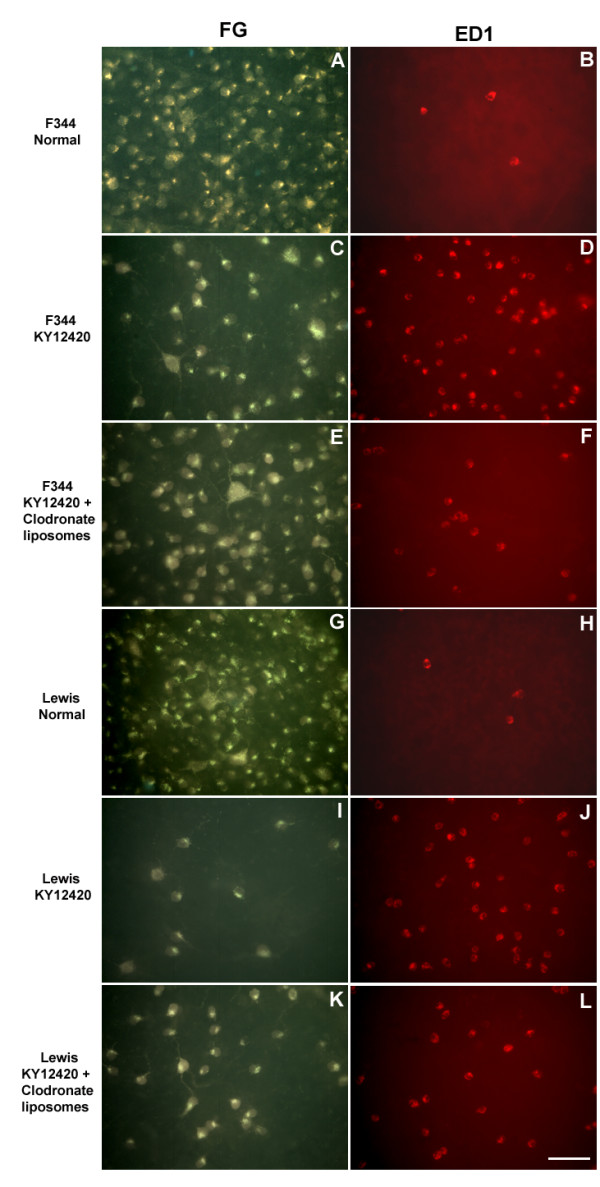
**Representative fluorescent photomicrographs of retinal wholemounts showing characteristics of retrogradely FG-labeled surviving RGCs (left column) and ED1^+ ^macrophages (right column).** A and B, normal F344 rat; C and D, after application of pathway inhibitor KY12420 in IOP-elevated F344 rat; E and F, after application of pathway inhibitor KY12420 and clodonate liposomes in IOP-elevated F344 rat; G and H, normal Lewis rat; I and J, after application of pathway inhibitor KY12420 in IOP-elevated Lewis rat; K and L, after application of pathway inhibitor KY12420 and clodonate liposomes in IOP-elevated Lewis rat. More surviving RGCs were seen in F344 than Lewis rats after IOP elevation and pathway inhibition (C versus I). After removal of macrophages by clodronate liposomes, the numbers of surviving RGCs increased in both F344 (E versus C) and Lewis (K versus I) rats. Similar results were obtained after inhibition of JAK/STAT pathway (images not shown). Scale bar = 50 μm.

Representative photomicrographs showing the appearances of FG-labelled viable RGCs (left column) and ED1^+ ^macrophages (right column) in normal F344 and Lewis rats or 3 weeks after acute IOP elevation plus various experimental interventions are shown in Figure 1. The appearances of FG-labelled viable RGCs 3 weeks after acute IOP elevation alone in both F344 and Lewis rats were previously shown [17]. Compared with normal intact F344 (A) and Lewis (G) rats, significant loss of RGCs was seen 3 weeks after acute IOP elevation and pathway inhibition of PI3K/akt in both strains (C and I, respectively). Clodronate liposomes significantly reduced the number of macrophages in the eye of both strains and, compared with the same strain of rats not receiving clodornate liposomes, significantly enhanced the number of surviving RGCs. However, the extent of surviving RGCs after macrophage removal was still below the intact control, especially in Lewis rats. Similar observations were seen after PI3K/akt pathway inhibition by LY294002 or JAK/STAT pathway inhibition by AG490 and Jak Inhibitor I (images not shown). More surviving RGCs were seen in the central than the peripheral region. No clear change in the retinal thickness was observed before and after the acute IOP elevation [27].

**Figure 2:**
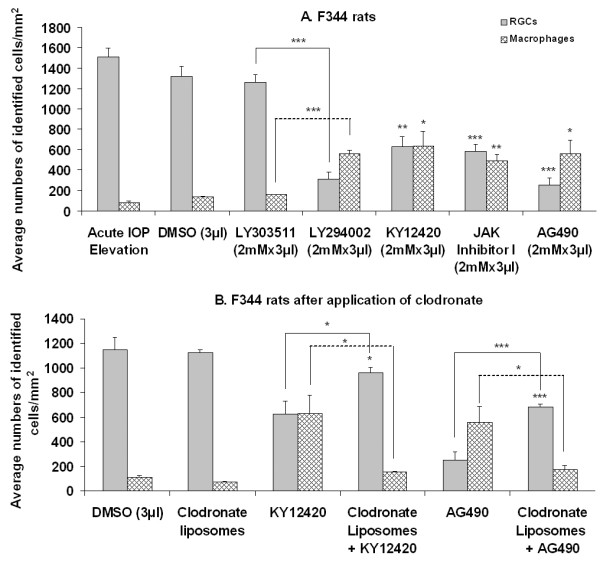
**Average densities (± SEM) of FG-labeled surviving RGCs and ED1^+ ^macrophages after inhibition of PI3K/akt and JAK/STAT pathways in F344 rats.** Significant decrease in RGC viability and concomitant increase in the number of macrophages were seen following PI3k/akt and JAK/STAT pathway inhibition (A). After macrophage removal (B), pathway inhibition-induced RGC loss was significantly but not completely prevented, indicating that both macrophage and PI3k/akt and JAK/STAT pathways were involved in RGC viability in this strain of rat. *p < 0.05, **p < 0.01 and ***p < 0.001 against DMSO group unless specified.

The average numbers ± SEM of surviving RGCs and ED1^+ ^macrophages were 2356 ± 125/retina and 11 ± 2/retina, respectively, in intact F344 rats (n = 4). The average numbers ( ± SEM) of surviving RGCs in the retinas of the IOP elevation only group (n = 6) and the inhibitor carrier DMSO group (n = 5) were not significantly different from each other (1514 ± 78/retina versus 1314 ± 103/retina; Fig. 2A). Intravitreal application of DMSO marginally increased the number of macrophages (81 ± 10/retina versus 137 ± 9/retina) in the eye (Fig. 2A). LY303511, the negative control of LY294002 and which contains a single atom substitution in the morpholine ring compared to LY294002 and does not inhibit PI3K even at high concentrations, did not affect RGC survival, but substantially increased the number of macrophages in the eye (n = 5; Fig. 2A). Compared with the negative control LY303511 and DMSO groups, significant decreases in RGC survival were observed after intravitreal application of PI3K/akt pathway inhibitor LY294002 or KY12420 (n = 5 in each group; Fig. 2A). Concomitant with the decrease in RGC survival was a significant increase in the number of macrophages in the retina (Fig. 2A). Significant decrease in the number of surviving RGCs was also seen after intravitreal application of JAK/STAT pathway inhibitor AG490 or Jak Inhibitor I (n = 5 and 6, respectively; Fig. 2A). Accompanying the decrease in RGC survival was a significant increase in the number of macrophages in the retina (Fig. 2A). These observations are thus similar to what was seen after inhibition of PI3K/akt and JAK/STAT pathways in SPD rats [26,27]. Our earlier studies using Western blotting showed that the pathway inhibitors used at these concentrations effectively, though not completely, blocked collective signal transduction of the pathways [26-28].

### Effects of macrophage removal on RGC viability in F344 rats

We applied a macrophage remover, clodronate liposomes, intravitreally to deplete macrophages in the eye [26-28,31]. As expected, clodronate liposomes applied alone or in combination with KY12420 (n = 5 each group) significantly reduced the number of macrophages in the eye to a level slightly higher than that in DMSO group (Fig. 2B). Concomitant with this reduction of macrophages in the eye was significant improvement but not complete recovery in RGC survival in KY12420-treated F344 rats (Fig. 2B). Similarly, an increased number of surviving RGCs was also seen after co-application of clodronate liposomes with JAK/STAT pathway inhibitor AG490 (n = 5; Fig. 2B). The improvement but not complete recovery of RGC survival after macrophage removal in PI3K/akt or Jak/STAT pathway-inhibited eyes suggested that macrophages and the pathways modulated, in opposite directions, RGC survival after acute IOP elevation in F344 rats.

### Effect of inhibition of PI3K/akt and JAK/STAT pathways on RGC viability in Lewis rats

**Figure 3:**
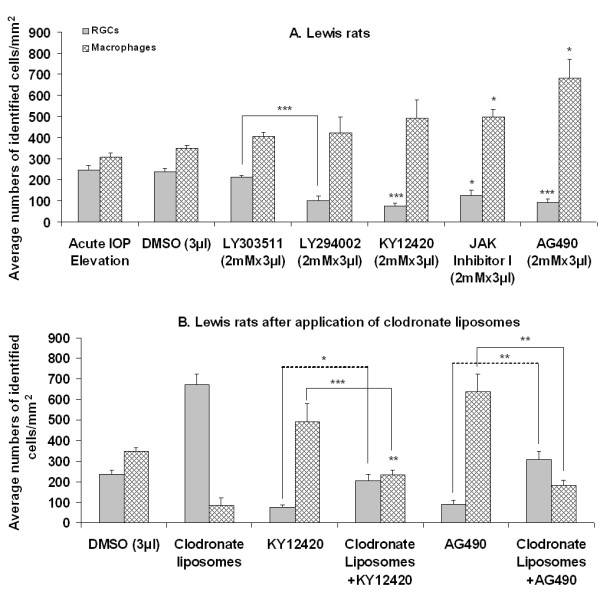
**Average densities (± SEM) of FG-labeled surviving RGCs and ED1^+ ^macrophages after inhibition of PI3K/akt and JAK/STAT pathways in Lewis rats.** Significant decrease in RGC viability and concomitant increase in the number of macrophages were seen following PI3k/akt and JAK/STAT pathway inhibition (A). After macrophage removal (B), pathway inhibition-induced RGC loss was significantly but not completely prevented, indicating that both macrophage and the pathways were also involved in RGC viability in Lewis rats. Note that the numbers of surviving RGCs were significantly lower in Lewis than F344 rats. *p < 0.05, **p < 0.01 and ***p < 0.001 against DMSO group unless specified.

The average numbers ± SEM of surviving RGCs and ED1^+ ^macrophages were 2583 ± 82/retina and 8 ± 1/retina, respectively, in intact Lewis rats (n = 4). The average numbers ( ± SEM) of surviving RGCs and ED1^+^macrophages in the retinas of IOP elevation only (n = 5) and DMSO (n = 4) groups were not significantly different from each other in Lewis rats (Fig. 3A). However, the number of surviving RGCs 3 weeks after acute IOP elevation was significantly (6-fold) lower in Lewis than F344 rats (Fig. 2A and 3A). LY303511 also did not affect RGC survival although it marginally increased the number of macrophages in Lewis rats (n = 5; Fig. 3). Even though the levels of RGC survival were already low in the negative control and DMSO groups (Fig. 3A), further decrease in RGC survival was still seen after intravitreal application of PI3K/akt pathway inhibitor LY294002 or KY12420 (n = 5 each group). Concomitant with the decrease in RGC survival was a significant increase in the number of macrophages in the retina (Fig. 3A). Similarly, intravitreal applications of JAK/STAT pathway inhibitor AG490 or Jak Inhibitor I (n = 5 each group) also resulted in a significant decrease in the number of surviving RGCs and a significant increase in the number of macrophages in the retina (Fig. 3).

### Effects of macrophage removal on RGC viability in Lewis rats

Similar to what occurred in F344 rats, clodronate liposomes applied alone (n = 5) or co-applied with KY12420 (n = 4) or AG490 (n = 5) significantly reduced the number of macrophages in the retina to a level lower than that in the DMSO group (Fig. 3B). Concomitant with the reductions of macrophages in the eye was significant improvement but not complete recovery in RGC survival in KY12420- and AG490-treated Lewis rats (Fig. 3B). These findings suggested that PI3K/akt and JAK/STAT pathways and macrophages are involved in RGC viability in similar fashion to that in F344 rats following acute IOP elevation.

To clarify whether the observed reduction of RGC counts after inhibitor application resulted from interfered transport of FG, we carried out another experiment in which both FG and immunohistochemical approaches were used to label RGCs of the same retinas and the numbers of RGCs between the 2 approaches were compared. Compared with the number of TUJ1-immunostained RGCs of the same retinas, there were an average of 15% (n = 3) decrease and 12% (n = 3) increase in the numbers of FG-labeled RGCs in KY12420 and AG490 treatment groups, repsectively. The small differences between the 2 labelling approaches are not statistically significant, indicating that the intravitreal application of the pathway inhibitors does not influence the efficacy of the FG transport. Note that FG was applied 20 hours whereas the data presented below were obtained from rats that received FG 5 days after the administration of the pathway inhibitors. The further delayed application of FG rendered the inhibitors less likely to affect the retrograde transport of FG.

## Discussion

In this study we investigated the roles of PI3K/akt and JAK/STAT pathways and macrophages in RGC viability after acute IOP elevation in F344 and Lewis rats, which are known to have different autoimmune profiles. We show that in both rat strains, inhibition of PI3K/akt or JAK/STAT pathway reduces RGC survival and activates macrophages in the eye. In addition, both macrophage activation and PI3K/akt and JAK/STAT pathways mediate RGC viability, in opposite directions, after acute IOP elevation.

PI3K/akt and JAK/STAT are known to be the major signalling executors for neuronal survival [19,20,24,25,32-36]. Whereas PI3K/akt is the common signal transduction pathway underlying neurotrophin-induced biological actions, JAK/STAT is well-documented to be responsible for cytokine-induced effects [37]. Previously we showed that the pathway inhibitors applied at dosages as in this study significantly inhibited signal transduction of the respective pathways [26-28,38]. The clear differences in RGC survival or in macrophage recruitment between eyes treated with LY294002 and with its negative control LY303511 following IOP elevation in both F344 and Lewis rats are similar to what was seen in SPD rats [26,27]. These results confirmed that the actions of LY294002 on RGC viability and macrophage recruitment are dependent on PI3k/akt pathway-inhibition. The persistent loss of RGCs after PI3K/akt or JAK/STAT pathway inhibition in the absence of ocular macrophages (following clodronate liposome application) in both strains further verify that these signal transduction pathways mediate RGC survival following acute IOP elevation *independent of *influence of autoimmune background.

Previously T-cells were shown to play a part in differential protection of RGCs following episcleral and limbal vein cauterization-induced IOP elevation in F344 and Lewis rats [14]. In our earlier study, we showed that macrophage reactions to acute IOP elevation were also different in rats with different autoimmune backgrounds [17]. In the present study we demonstrated that in contrast to the actions on macrophages, autoimmune background did not modulate signal transduction pathways of PI3K/akt and JAK/STAT in RGC survival.

## Conclusion

PI3K/akt and JAK/STAT pathway mediate RGC survival after acute IOP elevation and autoimmune background does not influence the functional roles of these pathways. In addition, PI3K/akt and JAK/STAT pathway inhibition-induced macrophage activation in the eye is detrimental to RGCs following acute IOP elevation.

## Methods

A total number of 108 young adult (8–10 weeks old) F344 and Lewis rats were used, and each experimental group consisted of 4–6 rats. All experiments conformed to The Chinese University of Hong Kong Animal Experimentation Ethic Committee (AEEC) guidelines and were approved by the AEEC. All possible measures were taken to minimize suffering and limit the number of rats used in this study. All surgery was carried out under anaesthesia with a 1:1 mixture (1.5 ml/kg) of ketamine (100 mg/ml) and xylazine (20 mg/ml).

### Acute IOP elevation and the experimental groups

The acute IOP elevation procedure has previously been reported [39]. Briefly, a 27-gauge needle was placed in the anterior chamber of the left eye. The needle was connected to a container carrying 500 ml sterile normal saline. The container was raised to a height of 1496 mm above the eye to elevate the IOP to 110 mmHg for 2 hrs. Each strain of rats was allocated to different experimental groups after acute IOP elevation. The first group received no intravitreal injections and served as intact controls. The second group received intravitreal injections of DMSO (3 μl each injection), which is the inhibitor carrier. The third group received intravitreal injections of LY303511 (Calbiochem, San Diego, USA; 2 mM × 3 μl), which was the negative control of PI3k/akt pathway inhibitor LY294002. The fourth and fifth groups received intravitreal injections of PI3k/akt pathway inhibitors LY294002 [2-(4-Morpholinyl)-8-phenyl-1(4H)-benzopyran-4-one hydrochloride, Sigma; 2 mM × 3 μl and KY12420 (Calbiochem, San Diego, USA; 2 mM × 3 μl), respectively. The sixth and seventh groups received intravitreal injections of JAK/STAT pathway inhibitors AG490 [α-Cyano-(3,4-dihydroxy)-N-benzylcinnamide, Calbiochem; USA; 2 mM × 3 μl] and Jak Inhibitor I [2-(1,1-Dimethylethyl)-9-fluoro-3,6-dihydro-7H-benz[h]-imidaz [4,5-f]isoquinolin-7-one; Calbiochem; 2 mM × 3 μl], respectively. Two inhibitors of each pathway, coupled with the available negative control, LY303511, were used in aim to clarify or exclude the possibility of chemical effects of these inhibitors on RGC viability.

As macrophage activation has been seen to accompany acute IOP elevation and inhibition of PI3k/akt and JAK/STAT pathways, we used clodronate liposomes to remove macrophages in the eye and examined what roles macrophages and the two pathways played in RGC viability. In this part, 1 group received intravitreal injection (3 μl, once only) of clodronate liposomes immediately after acute IOP elevation, 2 groups received immediate intravitreal injection (3 μl, once only) of clodronate liposomes that were followed by 3 intravitreal injections of KY12420 and AG490, respectively, 3, 9 and 15 days later. Both clodronate and liposomes (if prepared of phosphatidylcholine and cholesterol) are not toxic. Liposome-encapsulated clodronate and liposomes containing PBS only (control liposomes) were prepared as previously described [40]. Clodronate was a gift of Roche Diagnostics GmbH (Mannheim, Germany). Phosphatidylcholine was obtained from Lipoid GmbH (Ludwigshafen, Germany), and cholesterol purchased from Sigma. Recently we showed that both PBS liposomes and clodronate liposomes applied at this dosage into the eye were not detrimental to RGCs [28,31]. All rats survived for 3 weeks after acute IOP elevation.

For intravitreal injections of DMSO or the inhibitors, each rat received three posterior chamber eye injections on days 3, 9 and 15 after IOP elevation. Intravitreal injection of 3 μl clodronate liposomes was carried out once only on the day of IOP elevation, which was 3 days prior to pathway inhibitor injection. For the eye injection, the micropipette was deliberately angled to avoid damage to the lens [41].

### Retrograde labeling of viable RGCs

To retrogradely label viable RGCs, a small piece of gelfoam soaked with 4% FluoroGold (FG, Fluorochrome Inc, Denver, USA) was applied at the newly cut stump of the proximal ON to retrogradely label viable RGCs. Animals survived for another 40 hours to maximize retrograde transport of the dye. Note that RGCs only start to die 5 days after ON axotomy in adult rats [42]. While deeply anaesthetized, the rats were perfused with cold 4% paraformaldehyde in phosphate buffer (0.1 M, pH 7.4). After dissection from the eye-cups, the retinas were post-fixed in 4% paraformaldehyde for 45 min, flat-mounted and temporarily coverslipped in anti-fading medium (Dako Corporation, Carpinteria, CA, USA). The number of FG-labelled RGCs in each field (0.25 × 0.25 mm^2^), sampled at a fixed distance from one another and in a pattern of grid intersections, was counted throughout the whole retina. A total of 70–80 fields, about 8–10% of the total retinal area, were sampled per retina. The average density of viable RGCs was obtained. This approach avoided problems associated with uneven distribution of RGCs in the retina.

In a separate experiment, we examined whether intravitreal application of the pathway inhibitors influenced the retrograde transport of FG. One inhibitor of each pathway (AG490 for JAK/STAT pathway and KY12420 for PI3K/akt pathway) was injected intravitreally into eyes of normal rats (n = 3 each group). FG was applied in the same way as above 20 hours after the inhibitor application, and the rats were killed 40 hours after FG application. After counting the number of FG-labeled RGCs, the retinas were immunostained for RGCs using the TUJ1 monoclonal antibody (against βIII-tubulin, BabCO, Richmond, CA, USA). βIII-tubulin has been shown to be an RGC-specific marker in retinal wholemounts [15,16,28,31,38,43,44].

### Immunohistochemical staining of macrophages

After counting the number of FG-labeled RGCs, retinas were used for immunostaining of macrophages. The retinas were thoroughly washed with PBS and blocked with 10% normal goat serum (NGS), 1% bovine serum albumin (BSA) and 0.2% Triton for 1 hour. They were then immunostained overnight at 4°C with ED1 antibody (1:200, Serotec, Oxford, UK) [15,28], were rinsed with PBS and incubated with conjugated Cy3 (Jackson ImmunoResearch Laboratories, West Grove, PA, USA; 1:400) secondary antibody overnight at 4°C. After 3 washes each for 5 minutes, the retinas were mounted with anti-fading fluorescence mounting medium and examined under the fluorescent microscope. ED1 positive (^+^) cells were counted in the same way as for FG-labeled RGCs.

### Statistical analysis

Data on RGCs from the different groups were pooled and statistically analysed using Bonferroni test following one-way analysis of variance (ANOVA). Bonferroni test was used to compare mean values among all intra-groups [15,38].

## Abbreviations

EAE: experimental autoimmune encephalomyelitis; F344: Fischer 344; HPA: hypothalamic-pituitary-adrenal; IOP: intraocular pressure; GCL: ganglion cell layer; JAK: janus kinases; ON: optic nerve; PI3K: phosphatidylinositol 3-kinase; RGC: retinal ganglion cell; SPD: Sprague Dawley; STAT: signal transducers and activators of transcription.

## Authors' contributions

YH carried out experiments and collected data. ZL carried out the double-labeling experiment. NW participated in the study design. NvR provided reagents (clodronate liposomes). QC designed the study, analyzed the data and drafted the manuscript. All authors read and approved the final manuscript.

## References

[B1] Osborne NN, Casson RJ, Wood JP, Chidlow G, Graham M, Melena J (2004). Retinal ischemia: mechanisms of damage and potential therapeutic strategies. Prog Retin Eye Res.

[B2] Buchi ER (1992). Cell death in the rat retina after a pressure-induced ischaemia-reperfusion insult: an electron microscopic study. I. Ganglion cell layer and inner nuclear layer. Exp Eye Res.

[B3] Pease ME, McKinnon SJ, Quigley HA, Kerrigan-Baumrind LA, Zack DJ (2000). Obstructed axonal transport of BDNF and its receptor TrkB in experimental glaucoma. Invest Ophthalmol Vis Sci.

[B4] Martin KR, Quigley HA, Valenta D, Kielczewski J, Pease ME (2006). Optic nerve dynein motor protein distribution changes with intraocular pressure elevation in a rat model of glaucoma. Exp Eye Res.

[B5] Oka T, Tamada Y, Nakajima E, Shearer TR, Azuma M (2006). Presence of calpain induced proteolysis in retinal degeneration and dysfunction in a rat model of acute ocular hypertension. J Neurosci Res.

[B6] Blight AR (1992). Macrophages and inflammatory damage in spinal cord injury. J Neurotrauma.

[B7] Dijkstra CD, de Groot CJ, Huitinga I (1992). The role of macrophages in demyelination. J Neuroimmunol.

[B8] Huitinga I, Van Rooijen N, De Groot CJ, Uidehaag BM, Dijkstra CD (1990). Suppression of experimental allergic encephalomyelitis in Lewis rats after elimination of macrophages. J Exp Med.

[B9] Huitinga, Ruuls ISR, Jung S, Van Rooijen N, Hartung HP, Dijkstra CD (1995). Macrophages in T cell line-mediated, demyelinating, and chronic relapsing experimental autoimmune encephalomyelitis in Lewis rats. Clin Exp Immunol.

[B10] Hirschberg DL, Yoles E, Belkin M, Schwartz M (1994). Inflammation after axonal injury has conflicting consequences for recovery of function: Rescue of spared axons is impaired but regeneration is supported. J Neuroimmunol.

[B11] Popovish PG, Stokes BT, Whitacre CC (1996). Concept of autoimmunity following spinal cord injury: possible roles for T lymphocytes in the traumatized central nervous system. J Neurosci Res.

[B12] Newman TA, Woolley ST, Hughes PM, Sibson NR, Anthony DC, Perry VH (2001). T-cell- and macrophage-mediated axon damage in the absence of a CNS-specific immune response: involvement of metalloproteinases. Brain.

[B13] Neumann H (2003). Molecular mechanisms of axonal damage in inflammatory central nervous system diseases. Curr Opin Neurol.

[B14] Bakalash S, Kipnis J, Yoles E, Schwartz M (2002). Resistance of retinal ganglion cells to an increase in intraocular pressure is immune-dependent. Invest Ophthalmol Vis Sci.

[B15] Yin Y, Cui Q, Li Y, Irwin N, Fischer D, Harvey AR, Benowitz LI (2003). Macrophage-derived factors stimulate optic nerve regeneration. J Neurosci.

[B16] Luo JM, Zhi Y, Chen Q, Cen LP, Zhang CW, Lam DSC, Harvey AR, Cui Q (2007). Influence of macrophages and lymphocytes on the survival and axon regeneration of injured retinal ganglion cells in rats from different autoimmune backgrounds. Eur J Neurosci.

[B17] Huang Y, Li Z, van Rooijen N, Wang N, Pang CP, Cui Q (2007). Different responses of macrophages in retinal ganglion cell survival after acute ocular hypertension in rats with different autoimmune backgrounds. Exp Eye Res.

[B18] Kermer P, Klöcker N, Labes M, Bähr M (2000). Insulin-like growth factor-I protects axotomised rat retinal gnalgion cells from secondary death via PI3-K-dependent Akt phosphorylation and inhibition of caspase-3 *in vivo*. J Neurosci.

[B19] Alonzi T, Middleton G, Wyatt S, Buchman V, Betz UA, Müller W, Musiani P, Poli V, Davies AM (2001). Role of STAT3 and PI 3-kinase/Akt in mediating the survival actions of cytokines on sensory neurons. Mol Cell Neurosci.

[B20] Dolcet X, Soler RM, Gould TW, Egea J, Oppenheim RW, Comella JX (2001). Cytokines promote motoneuron survival through the janus kinase-dependent activation of the phosphatidylinositol 3-kinase pathway. Mol Cell Neurosci.

[B21] Cheng L, Sapieha P, Kittlerová P, Hauswirth WW, Di Polo A (2002). TrkB gene transfer protects retinal ganglion cells from axotomy-induced death *in vivo*. J Neurosci.

[B22] Kretz A, Happold CJ, Marticke JK, Isenmann S (2005). Erythropoietin promotes regeneration of adult CNS neurons via Jak2Stat3 and PI3K/AKT pathway activation. Mol Cell Neurosci.

[B23] Kretz A, Schmeer C, Tausch S, Isenmann S (2006). Simvastatin promotes heat shock protein 27 expression and Akt activation in the rat retina and protects axotomized retinal ganglionc cells *in vivo*. Neurobiol Dis.

[B24] Yadav A, Kalita A, Dhillon S, Banerjee K (2005). JAK/STAT3 pathway is involved in survival of neurons in response to insulin-like growth factor and negatively regulated by suppressor of cytokine signalling-3. J Biol Chem.

[B25] Yamauchi K, Osuka K, Takayasu M, Usuda N, Nakazawa A, Nakahara N, Yoshida M, Aoshima C, Hara M, Yoshida J (2006). Activation of JAK/STAT signalling in neurons following spinal cord injury in mice. J Neurochem.

[B26] Huang Y, Cen LP, Choy KW, van Rooijen N, Wang N, Pang CP, Cui Q (2007). JAK/STAT pathway mediates retinal ganglion cell survival after acute ocular hypertension but not under normal conditions. Exp Eye Res.

[B27] Huang Y, Cen LP, Luo JM, Wang NL, Zhang MZ, van Rooijen N, Pang CP, Cui Q (2008). Differential Roles of PI3K/akt Pathway in Retinal Ganglion Cell Survival in Rats with or without Acute Ocular Hypertension. Neurosci.

[B28] Luo JM, Cen LP, Zhang XM, Chiang SWY, Huang Y, Lin D, Fan Y, van Rooijen N, Lam DSC, Pang CP, Cui Q (2007). PI3K/akt, JAK/STAT and MEK/ERK pathway inhibition protects retinal ganglion cells via different mechanisms after optic nerve injury. Eur J Neurosci.

[B29] Wilder RL, Griffiths MM, Cannon GW, Gaspi R, Remmers EF (2000). Susceptibility to autoimmune disease and drug addiction in inbred rats. Are there mechanistic factors in common related to abnormalities in hypothalamicepituitaryeadrenal axis and stress response function?. Ann NY Acad Sci.

[B30] Sun D, Whitaker JN, Wilson DB (1999). Regulatory T cells in experimental allergic encephalomyelitis. III. Comparison of disease resistance in Lewis and Fischer 344 rats. Eur J Immunol.

[B31] Cen LP, Luo JM, Zhang CW, Fan YM, Song Y, So KF, van Rooijen N, Pang CP, Lam DS, Cui Q (2007). Chemotactic effect of ciliary neurotrophic factor on macrophages in retinal ganglion cell survival and axonal regeneration. Invest Ophthalmol Vis Sci.

[B32] Crowder RJ, Freeman RS (1999). The survival of sympathetic neurons promoted by potassium depolarization, but not by cyclic AMP, requires phosphatidylinositol 3-kinase and Akt. J Neurochem.

[B33] Datta SR, Brunet A, Greenberg M (1999). Cellular survival: a play in three Akts. Genes Dev.

[B34] Klocker N, Kermer P, Weishaupt JH, Labes M, Ankerhold R, Bahr M (2000). Brain-derived neurotrophic factor-mediated neuroprotection of adult rat retinal ganglion cells *in vivo* does not exclusively depend on phosphatidyl-inositol-3'-kinase/protein kinase B signalling. J Neurosci.

[B35] Brunet A, Datta SR, Greenberg ME (2001). Transcription-dependent and -independent control of neuronal survival by the PI3K-Akt signalling pathway. Curr Opin Neurobiol.

[B36] Rong R, Ahn J-Y, Huang H, Nagata E, Kalman D, Kapp JA, Tu J, Worley PF, Snyder SH, Ye K (2003). PI3 Kinase enhancer-Homer complex couples mGluR1 to PI3 kinase, precenting neuronal apoptosis. Nat Neurosci.

[B37] O'Shea JJ, Gadina M, Schreiber RD (2002). Cytokine signaling in 2002: new surprise in the Jak/Stat pathway. Cell.

[B38] Park K, Luo JM, Hisheh S, Harvey AR, Cui Q (2004). Cellular mechanisms associated with spontaneous and ciliary neurotrophic factor-cAMP-induced survival and axonal regeneration of adult retinal ganglion cells. J Neurosci.

[B39] Roth S, Shaikh AR, Hennelly MM, Li Q, Bindokas V, Graham CE (2003). Mitogen-activated protein kinases and retinal ischemia. Invest Ophthalmol Vis Sci.

[B40] Van Rooijen N, Sanders A (1994). Liposome mediated depletion of macrophages: Mechanism of action, preparation of liposomes and applications. J Immunol Meth.

[B41] Leon S, Yin Y, Nguyen J, Irwin N, Benowitz LI (2000). Lens injury stimulates axon regeneration in the mature rat optic nerve. J Neurosci.

[B42] Berkelaar M, Clarke DB, Wang YC, Bray GM, Aguayo AJ (1994). Axotomy results in delayed death and apoptosis of retinal ganglion cells in adult rats. J Neurosci.

[B43] Fischer D, He Z, Benowitz LI (2004). Counteracting the Nogo receptor enhances optic nerve regeneration if retinal ganglion cells are in an active growth state. J Neurosci.

[B44] Koprivica V, Cho KS, Park JB, Yiu G, Atwal J, Gore B, Kim JA, Lin E, Tessier-Lavigne M, Chen DF, He Z (2005). EGFR activation mediates inhibition of axon regeneration by myelin and chondroitin sulfate proteoglycans. Science.

